# Supplementary material for: An Ontological Framework to Facilitate Early Detection of ‘Radicalization’ (OFEDR)—A Three World Perspective
Source: J Imaging. 2021 Mar 22;7(3):60. doi: 10.3390/jimaging7030060 (PMC8321290; doi:10.3390/jimaging7030060)
Supplement: Supplementary file 1 [file jimaging-07-00060-s001.zip › jimaging-1113344 final TBS/jimaging-1113344 final/Supplementary.pdf]

Supplementary Materials: An Ontological Framework to Facilitate Early Detection of 'Radicalization' (OFEDR) –A Three World Perspective

Linda Wendelberg * [1,‡] https://orcid.org/0000-0002-1339-469X

Results from Literature Search

Table S1: A non-exhaustive example of sources, which may be of relevance for development of an ontology about IBBB's for EA and radicalization.

Name	Description	Type
NBO [1]	Neuroscience	Ontology
Allen Brain Map [2]	Neuroscience	Ontology
Geneontology [3,4]	Genes	Ontology
Varoquaux, 2018 [5]	Cognitive	Ontology
	Brain Mapping	
Monarch Initiative [6,7]	Phenotype	Ontology
EMBL [8]	Human	Ontology
	Disease	
MFOEM [9]	Emotion	Ontology
MF [10]	Cognition Traits	Ontology
	Disease Disorders	
ONL-MSA [11]	Mental State	Ontology
	Assessment	
NIO [12]	Neuropsychology	Ontology
	Brain Area	
	Cognitive	
APAONTO [13]	Pscyhology	Ontology
Etudo, 2017 [?]	Terrorism	Ontology
		system
BrainMap [14,15]	Neuroscience	Database
GTD [16,17]	Terrorism	Database
Poldrack, 2016 [18]	Cognitive	Article
Haendel, 2018 [19]	Medicine	Article
Barhamgi, 2018 [20]	Radicalization	Article
	Social Networks	
Barhamgi, 2018 [21]	Radicalization	Article
	Social Networks	Article
Rodrigues, 2014 [22]	Behavior	Article
Masmoudi, 2018 [23]	Radicalization	Article
	Online messages	
Mannes, 2005 [24]	Terrorism	Article
Galjano, 2009 [25]	Terrorism	Article
ICCT[26]	Counter-Terrorism	Think and
		do tank
BOCV [27]	Research Guide	Library
Perspectives on	Terrorism	Journal
Terrorism		
JD	Radicalization	Journal
Terror Polit Violenc.	Terrorism	Journal
Stud. Confl.	Terrorism	Journal
Terror.		
Crit Stud Terror	Terrorism	Journal
Behav. Sci. Terror.	Terrorism	Journal
Political Aggress.		
IJCWT	Terrorism	Journal
J. Polic. Intell.	Terrorism	Journal
Count. Terror.		

SPARQL Query Examples & Information about Test Samples

Table S2: SPARQL Queries. Values that are not from real data sample are assumed based on peer reviewed publications. *value also given to control group to increase difficulty

Query	Real Data Yes/No	Statistical About	Scientific Evidence	Modification Abstraction	New Score
**Model I**		No individuals from control group was detected			
?individual onto:hasSmallerPupil ?PupilDiameter .	Yes		Small	less insula activation?[29]	Medium
?individual onto:hasShorterFixFetishStimuli ?fixationShort .	Yes		Small	different profiles for sex[28]	medium
?individual onto:hasLongFixAllStimuli ?fixationLong .	Yes		Small	depend on stimuli if familiarity	Moderate/ Large
?individual onto:hasAmbientChangeToPositive ?Ambient .	Yes		Small	few studies with evidence acting out but exist	small
?individual onto:hasFocalChangeToNegative ?Focal .	Yes		Small	can relate to sexual change=evidence-high	Large
?individual onto:hasRadicaleValuesBeliefs ?ValuesBeliefs*	No		Large	High evidence	Very/ Large
**Model II**		No individuals from control group was detected			
?individual onto:hasRadicaleValuesBeliefs ?ValuesBeliefs .	No		Large	high evidence	Large Very Large
?individual onto:hasSmallerPupil ?PupilDiameter .	Yes		Small	less insula activation?[29]	Medium
?individual onto:hasAmbientChangeToPositive ?Ambient .	Yes		Small	few studies evidence-acting out but exist	small
?individual onto:hasFocalChangeToNegative ?Focal	Yes		Small	if use findings sexual change	Large
**Model III**		No individuals from control group was detected			
?individual onto:hasLowSelf_Esteem ?SelfEsteem.	Yes		Large	evidence high	Very Large
?individual onto:hasProlongAvrLatency ?Latency.	Yes		Medium	depend on stimuli type not use single variable	small
?individual onto:hasAge18-30 ?Age .	Yes		Medium	related to terrorism some evidence young higher sensitivity EA	Medium
?individual onto:hasSmallerPupil ?PupilDiameter .	Yes		Small	less insula activation?[29]	Medium
**Model IV**		No individuals from control group was detected			
?individual onto:hasSmallerPupil ?PupilDiameter .	Yes		Small	less insula activation?[29]	Medium Small
?individual onto:hasChangeSerotonin ?Serotonin .	No			few exact studies EA worsens psychiatric symptoms	Small
?individual onto:hasLowSelf_Esteem ?SelfEsteem .	Yes		Large	high evidence	Very Large
?individual onto:hasRedBodyAwareness ?BodyAwareness .	No		Medium	consistent with other evidence e.g., insula	High
**Model V**		No individuals from control group was detected			
?individual onto:hasSmallerPupil ?PupilDiameter .	Yes		Small	less insula activation?[29]	Medium
?individual onto:hasHighSelfEsteem ?SelfEsteem .	Yes		Medium	evidence high<low different pattern	Medium
?individual onto:hasHighExtraversion ?Extraversion .	Yes		Small	low evidence can be related to high self-esteem author has some evidence	Small
?individual onto:hasInhibition ?Inhibition .	No		Medium	important-predict overcontrol-more serious violence	Medium

Table S3: Participant characteristics. Pearson correlation coefficient.

Participant Characteristics	Experiment Mean/SD	Control Mean/SD	Test	Correlations*
**PANAS-Negative affect**	M=20.5 SD = 6.58	M = 19.1 SD = 5.96	(t(77)=-.99, p = 0.33)	$p < .01$ level: agreeableness -.32, neuroticism .34, self-esteem -.49
**PANAS-Positive affect**	M = 30.3 SD=6.7	M=30.67 SD =5.57	(t(77)=.33 p=0.33)	$p < .01$ level: extraversion .44, self-esteem .36
**Extraversion**	M=6.43 SD =1.95	M=6.78 SD=1.93	(t(78)=.81 p=.42)	$p < .01$ level: self-esteem .29
**Agreeableness**	M=7.1 SD=1.78	M=6.8 SD=1.5	(t(78)=-.75 p=.46)	$p < .05$ level: neuroticism-.27, self-esteem .26
**Conscientiousness**	M=7.0 SD = 1.6	M=7.2 SD=1.3	(t(78)=.46 p=.46)	$p < .05$ self-esteem .26
**Neuroticism**	M=6.28 SD=2.4	M=5.7 SD=1.67	(t(78)=-1.3 , p=.20)	$p < .05$ level: agreeableness-.27, $p < .01$ level: self-esteem -.42
**Openness to experience**	M=6.45 SD=2.46	M=7.05 SD=1.99	(t(78)=.39 p=.70)	No correlations
**Self-Esteem**	M=27.83 SD=4.68	M=28.73 SD=5.12	(t(78)=.82 p=.42)	Correlated with all variables except openness to experience

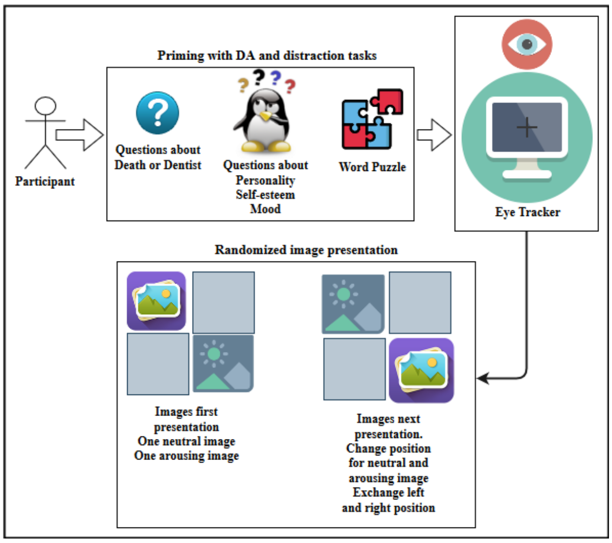

Figure S1. Visualization of data gathering procedure related to test data. Images were presented randomly on a display consisting of two images (one neutral, one fetish). It was controled for natural leftward bias.

References

1. BioPortal Neurobehavior Ontology. Available online:http://bioportal.bioontology.org /ontologies/NBO (accessed on 12.07.2020).
2. Allen Brain Atlas Dataportal. Available online:http://$help.brain - map.org/display/api/Atlas + Drawings + and + Ontologies$ (accessed on 12.07.2020).
3. Ashburner, M.; Ball, C. A.; Blake, J. A.; Botstein, D.; Butler, H.; Cherry, J. M.; Davis, A. P.; Dolinski, K.; Dwight, S. S.; Eppig, J. T.; Harris, M. A.; Hill, D. P.; Issel-Tarver, L.; Kasarskis, A.; Lewis, S.; Matese, J. C.; Richardson, J. E.; Ringwald, M.; Rubin, G. M.; Sherlock, G. Gene ontology: tool for the unification of biology. *Nat Genet.* **2000**, *25*, 25-29.
4. The Gene Ontology Consortium. The Gene Ontology Resource: 20 years and still GOing strong. *Nucleic Acids Res.* **2019** , *47*, 330-338.
5. Varoquaux, G.; Schwartz, Y.; Poldrack, R. A.; Gauthier, B.; Bzdok, D.; Poline J-B, et al. Atlases of Cognition with Large-Scale Human Brain Mapping. *PLoS Comput Biol.* **2018**, *14*, 1-18.
6. Köhler, S.; Carmody, L.; Vasilevsky, N.; Jacobsen, J. O. B. *et al.* Expansion of the Human Phenotype Ontology (HPO) knowledge base and resources. *Nucleic Acids Research.* **2019**, *47*, 1018-1027.
7. Human Phenotype Ontology (HPO). Available online: https://hpo.jax.org/app/ (accessed on 12.07.2020).
8. OLS Ontology Search Human Disease Ontology. Available online: https: *www.ebi.ac.ukolsontologiesdoid* (accessed on 12.07.2020).
9. Bioportal Emotion Ontology. Available online: http://bioportal.bioontology.org/ontologies/MFOEM (accessed on 12.07.2020).
10. Bioportal Mental Functioning Ontology Available online: http://bioportal.bioontology.org/ontologies/MF (accessed on 12.07.2020).
11. Bioportal Mental State Assessment. Available online: http://bioportal.bioontology.org/ontologies/ONL-MSA (accessed on 12.07.2020).
12. Bioportal Neuropsychological Integrative Ontology. Available online: http://bioportal.bioontology.org/ontologies/NIO (accessed on 12.07.2020).
13. Bioportal Psychology Ontology (APAONTO). Available online: https://bioportal.bioontology.org/ontologies/ APAONTO (accessed on 12.07.2020).
14. Laird, A. R.; Lancaster, J.J.; Fox, P.T. BrainMap.*Neuroinform* **2005**, *3*, 65-77.
15. brainmap.org Available online: https://www.brainmap.org/ (accessed on 12.07.2020).
16. Global Terrorism Database. Managed by the National Consortium for the Study of Terrorism and Responses to Terrorism (START). **2009-2019**. University of Maryland.
17. Global Terrorism Database. Available online: https://www.start.umd.edu/gtd/ (accessed on 12.07.2020).
18. Poldrack, R. A.; Yarkoni, T. From Brain Maps to Cognitive Ontologies: Informatics and the Search for Mental Structure. Annu Rev Psychol. **2016** , *67*, 587-612.
19. Haendel, M. A.; Chute, C. G.; Robinson, P. N. Classification, Ontology, and Precision Medicine. *N. Engl J Med.* **2018**, *379*, 1452-1462.
20. Barhamgi, M.; Masmoudi, A.; Lara-Carbrera, R. Camacho, D. Social networks data analysis with semantics: application to the radicalization problem. *J Amb Intel Hum Comp.* **2018**, 1-15.
21. Barhamgi M.; Lara-Cabrera R.; Benslimane D.; Camacho D. Ontology Uses for Radicalisation Detection on Social Networks. In *Intelligent Data Engineering and Automated Learning (IDEAL)* Yin H.; Camacho D.; Novais P.; Tallón-Ballesteros A. Eds.; Springer Cham: 2018; pp. 3-8.
22. Rodríguez, N. D. A.; Cuéllar, M. P.; Lilius, J.; Calvo-Flores, M. D. Survey on Ontologies for Human Behavior Recognition. *ACM Computing Surveys* **2014**, *46*, 1-33.
23. Masmoudi, A.; Barhamgi, M.; Faci, N.; Saoud, Z.; Belhajjame, K.; Benslimane, D.; Camacho, D. An Ontology-based Approach for Mining Radicalization Indicators from Online Messages. *IEEE 32nd International Conference on Advanced Information Networking and Applications (AINA)* IEEE: Cracow, Poland, 2018; pp. 609-616.
24. Mannes, A.; Golbeck, J. Building a Terrorism Ontology. *SWC Workshop on Ontology Patterns for the Semantic Web.* Springer: Galway, Ireland, **2005**, pp. 1-12.
25. Galjano, P.; Popovich, V. Theoretical Investigation of Terrorism. Ontology Development. In *Information Fusion and Geographic Information Systems. Lecture Notes in Geoinformation and Cartography* Popovich V.V.; Claramunt C.; Schrenk M.; Korolenko K.V. Eds.: Springer: Berlin, Heidelberg 2009; pp. 227-239.
26. The International Centre for Counter-Terrorism —The Hague (ICCT) Available online: https://icct.nl/ (accessed on 12.07.2020).
27. University of Michigan. Research Guide. Biomedical Ontologies and Controlled Vocabularies. Available online: https://guides.lib.umich.edu/ontology (accessed on 12.07.2020)
28. Birnbaum G.; Hirschberger G.; Goldenberg J. Desire in the face of death: Terror management, attachment, and sexual motivation. *Personal Relationship* **2011**, *18*, 1-19.
29. Kucyi, A.; Parvizi, J. Pupillary Dynamics Link Spontaneous and Task-Evoked Activations Recorded Directly from Human Insula. *J Neurosci.* **20**, *40*, 6207-6218.
