# Supplementary material for: An Ontological Framework to Facilitate Early Detection of ‘Radicalization’ (OFEDR)—A Three World Perspective
Source: J Imaging. 2021 Mar 22;7(3):60. doi: 10.3390/jimaging7030060 (PMC8321290; doi:10.3390/jimaging7030060)
Supplement: Supplementary file 1 [file jimaging-07-00060-s001.zip › jimaging-1113344 final TBS/jimaging-1113344 final/template.pdf]

MDPI

Article
Title

Firstname Lastname [1,†,‡], Firstname Lastname [1,‡] and Firstname Lastname [2,*]

[1] Affiliation 1; e-mail@e-mail.com
[2] Affiliation 2; e-mail@e-mail.com
* Correspondence: e-mail@e-mail.com; Tel.: (optional; include country code; if there are multiple corresponding authors, add author initials) +xx-xxxx-xxx-xxxx (F.L.)
† Current address: Affiliation 3
‡ These authors contributed equally to this work.

Abstract: A single paragraph of about 200 words maximum. For research articles, abstracts should give a pertinent overview of the work. We strongly encourage authors to use the following style of structured abstracts, but without headings: (1) Background: place the question addressed in a broad context and highlight the purpose of the study; (2) Methods: describe briefly the main methods or treatments applied; (3) Results: summarize the article's main findings; (4) Conclusion: indicate the main conclusions or interpretations. The abstract should be an objective representation of the article, it must not contain results which are not presented and substantiated in the main text and should not exaggerate the main conclusions.

Keywords: keyword 1; keyword 2; keyword 3 (List three to ten pertinent keywords specific to the article; yet reasonably common within the subject discipline.)

0. How to Use this Template

The template details the sections that can be used in a manuscript. Note that the order and names of article sections may differ from the requirements of the journal (e.g., the positioning of the Materials and Methods section). Please check the instructions on the authors' page of the journal to verify the correct order and names. For any questions, please contact the editorial office of the journal or support@mdpi.com. For LaTeX-related questions please contact latex@mdpi.com.

1. Introduction

The introduction should briefly place the study in a broad context and highlight why it is important. It should define the purpose of the work and its significance. The current state of the research field should be reviewed carefully and key publications cited. Please highlight controversial and diverging hypotheses when necessary. Finally, briefly mention the main aim of the work and highlight the principal conclusions. As far as possible, please keep the introduction comprehensible to scientists outside your particular field of research. Citing a journal paper [1]. Now citing a book reference [2,3] or other reference types [4–6]. Please use the command [7,8] for the following MDPI journals, which use author–date citation: Administrative Sciences, Arts, Econometrics, Economies, Genealogy, Histories, Humanities, IJFS, Journal of Intelligence, Journalism and Media, JRFM, Languages, Laws, Religions, Risks, Social Sciences.

2. Materials and Methods

Materials and Methods should be described with sufficient details to allow others to replicate and build on published results. Please note that publication of your manuscript implicates that you must make all materials, data, computer code, and protocols associated with the publication available to readers. Please disclose at the submission stage any restrictions on the availability of materials or information. New methods and

Citation: Lastname, F.; Lastname, F.; Lastname, F. Title. *Journal Not Specified* **2021**, *1*, 0. https://doi.org/

Received:
Accepted:
Published:

Publisher's Note: MDPI stays neutral with regard to jurisdictional claims in published maps and institutional affiliations.

Copyright: © 2021 by the authors. Submitted to *Journal Not Specified* for possible open access publication under the terms and conditions of the Creative Commons Attribution (CC BY) license (https://creativecommons.org/licenses/by/4.0/).

protocols should be described in detail while well-established methods can be briefly described and appropriately cited.

Research manuscripts reporting large datasets that are deposited in a publicly avail-able database should specify where the data have been deposited and provide the relevant accession numbers. If the accession numbers have not yet been obtained at the time of submission, please state that they will be provided during review. They must be provided prior to publication.

Interventionary studies involving animals or humans, and other studies require ethical approval must list the authority that provided approval and the corresponding ethical approval code.

> This is an example of a quote.

3. Results

This section may be divided by subheadings. It should provide a concise and precise description of the experimental results, their interpretation as well as the experimental conclusions that can be drawn.

3.1. Subsection

3.1.1. Subsubsection

Bulleted lists look like this:

- First bullet;
- Second bullet;
- Third bullet.

Numbered lists can be added as follows:

1. First item;
2. Second item;
3. Third item.

The text continues here.

3.2. Figures, Tables and Schemes

All figures and tables should be cited in the main text as Figure 1, Table A1, etc.

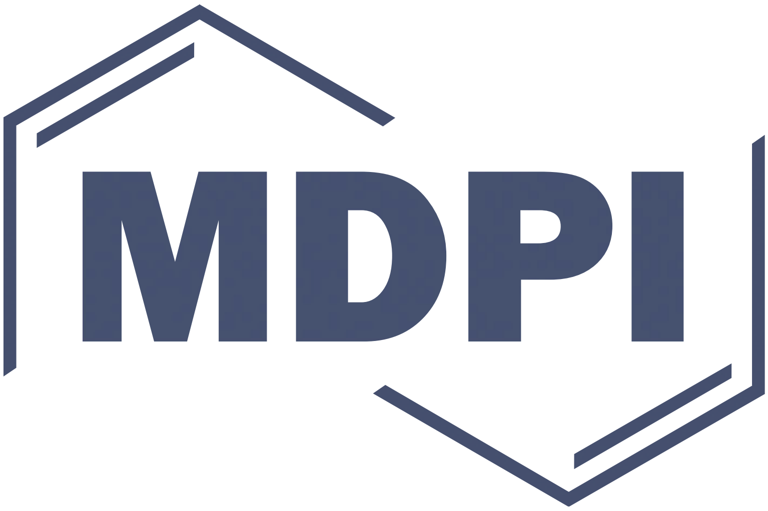

Figure 1. This is a figure. Schemes follow the same formatting. If there are multiple panels, they should be listed as: (**a**) Description of what is contained in the first panel. (**b**) Description of what is contained in the second panel. Figures should be placed in the main text near to the first time they are cited. A caption on a single line should be centered.

Table 1. This is a table caption. Tables should be placed in the main text near to the first time they are cited.

**Title 1**	**Title 2**	**Title 3**
Entry 1	Data	Data
Entry 2	Data	Data

Text.
Text.

3.3. Formatting of Mathematical Components

This is the example 1 of equation:

$$a = 1, \tag{1}$$

the text following an equation need not be a new paragraph. Please punctuate equations as regular text.

This is the example 2 of equation:

$$a = b + c + d + e + f + g + h + i + j + k + l + m + n + o + p + q + r + s + t + u + v + w + x + y + z \tag{2}$$

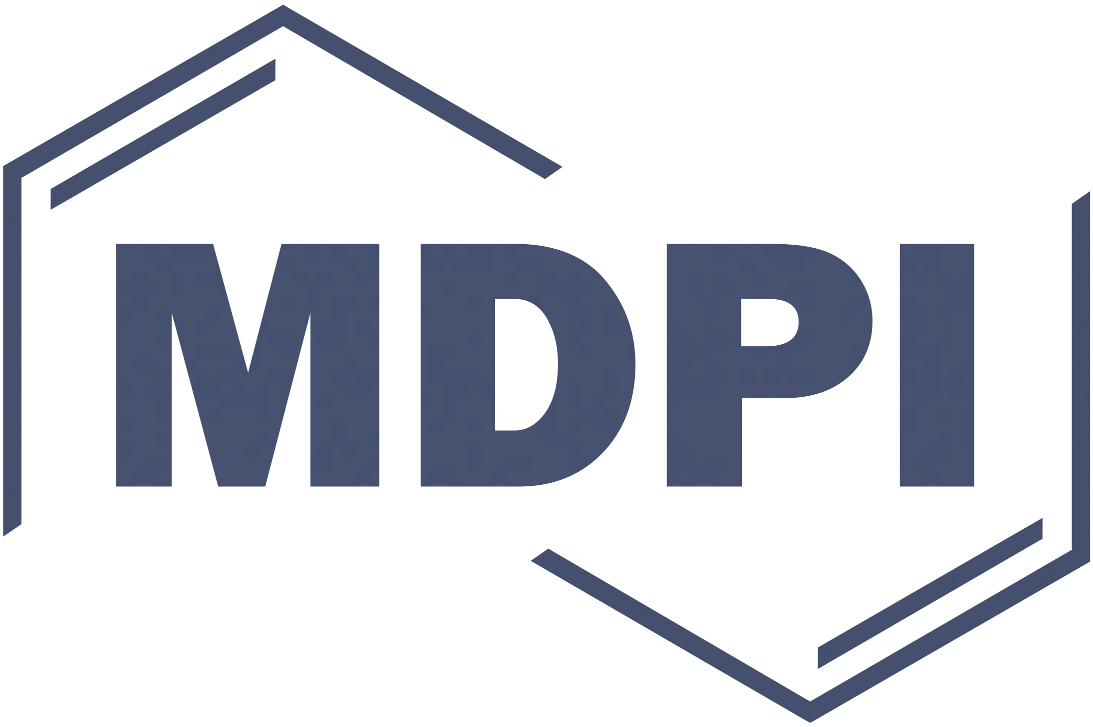

Figure 2. This is a wide figure.

Please punctuate equations as regular text. Theorem-type environments (including propositions, lemmas, corollaries etc.) can be formatted as follows:

Theorem 1. *Example text of a theorem.*

The text continues here. Proofs must be formatted as follows:

Proof of Theorem 1. Text of the proof. Note that the phrase "of Theorem 1" is optional if it is clear which theorem is being referred to. □

The text continues here.

4. Discussion

Authors should discuss the results and how they can be interpreted from the perspective of previous studies and of the working hypotheses. The findings and their implications should be discussed in the broadest context possible. Future research directions may also be highlighted.

5. Conclusions

This section is not mandatory, but can be added to the manuscript if the discussion is unusually long or complex.

6. Patents

This section is not mandatory, but may be added if there are patents resulting from the work reported in this manuscript.

Author Contributions: For research articles with several authors, a short paragraph specifying their individual contributions must be provided. The following statements should be used "Conceptualization, X.X. and Y.Y.; methodology, X.X.; software, X.X.; validation, X.X., Y.Y. and Z.Z.; formal analysis, X.X.; investigation, X.X.; resources, X.X.; data curation, X.X.; writing—original draft preparation, X.X.; writing—review and editing, X.X.; visualization, X.X.; supervision, X.X.; project administration, X.X.; funding acquisition, Y.Y. All authors have read and agreed to the published version of the manuscript.", please turn to the CRediT taxonomy for the term explanation. Authorship must be limited to those who have contributed substantially to the work reported.

Funding: Please add: "This research received no external funding" or "This research was funded by NAME OF FUNDER grant number XXX." and and "The APC was funded by XXX". Check carefully that the details given are accurate and use the standard spelling of funding agency names at https://search.crossref.org/funding, any errors may affect your future funding.

Institutional Review Board Statement: In this section, please add the Institutional Review Board Statement and approval number for studies involving humans or animals. Please note that the Editorial Office might ask you for further information. Please add "The study was conducted according to the guidelines of the Declaration of Helsinki, and approved by the Institutional Review Board (or Ethics Committee) of NAME OF INSTITUTE (protocol code XXX and date of approval)." OR "Ethical review and approval were waived for this study, due to REASON (please provide a detailed justification)." OR "Not applicable" for studies not involving humans or animals. You might also choose to exclude this statement if the study did not involve humans or animals.

Informed Consent Statement: Any research article describing a study involving humans should contain this statement. Please add "Informed consent was obtained from all subjects involved in the study." OR "Patient consent was waived due to REASON (please provide a detailed justification)." OR "Not applicable" for studies not involving humans. You might also choose to exclude this statement if the study did not involve humans.

Written informed consent for publication must be obtained from participating patients who can be identified (including by the patients themselves). Please state "Written informed consent has been obtained from the patient(s) to publish this paper" if applicable.

Data Availability Statement: In this section, please provide details regarding where data supporting reported results can be found, including links to publicly archived datasets analyzed or generated during the study. Please refer to suggested Data Availability Statements in section "MDPI Research Data Policies" at https://www.mdpi.com/ethics. You might choose to exclude this statement if the study did not report any data.

Acknowledgments: In this section you can acknowledge any support given which is not covered by the author contribution or funding sections. This may include administrative and technical support, or donations in kind (e.g., materials used for experiments).

Conflicts of Interest: Declare conflicts of interest or state "The authors declare no conflict of interest." Authors must identify and declare any personal circumstances or interest that may be perceived as inappropriately influencing the representation or interpretation of reported research results. Any role of the funders in the design of the study; in the collection, analyses or interpretation of data; in the writing of the manuscript, or in the decision to publish the results must be declared in this section. If there is no role, please state "The funders had no role in the design of the study; in the collection, analyses, or interpretation of data; in the writing of the manuscript, or in the decision to publish the results".

Sample Availability: Samples of the compounds ... are available from the authors.

Abbreviations

The following abbreviations are used in this manuscript:

MDPI	Multidisciplinary Digital Publishing Institute
DOAJ	Directory of open access journals
TLA	Three letter acronym
LD	Linear dichroism

Appendix A

Appendix A.1

The appendix is an optional section that can contain details and data supplemental to the main text—for example, explanations of experimental details that would disrupt the flow of the main text but nonetheless remain crucial to understanding and reproducing the research shown; figures of replicates for experiments of which representative data are shown in the main text can be added here if brief, or as Supplementary Data. Mathematical proofs of results not central to the paper can be added as an appendix.

Table A1. This is a table caption. Tables should be placed in the main text near to the first time they are cited.

Title 1	Title 2	Title 3
Entry 1	Data	Data
Entry 2	Data	Data

Appendix B

All appendix sections must be cited in the main text. In the appendices, Figures, Tables, etc. should be labeled, starting with "A"—e.g., Figure A1, Figure A2, etc.

References

1. Author 1, T. The title of the cited article. *Journal Abbreviation* **2008**, *10*, 142–149.
2. Author 2, L. The title of the cited contribution. In *The Book Title*; Editor1, F., Editor2, A., Eds.; Publishing House: City, Country, 2007; pp. 32–58.
3. Author 1, A.; Author 2, B. *Book Title*, 3rd ed.; Publisher: Publisher Location, Country, 2008; pp. 154–196.
4. Author 1, A.B.; Author 2, C. Title of Unpublished Work. *Abbreviated Journal Name* stage of publication (under review; accepted; in press).
5. Author 1, A.B. (University, City, State, Country); Author 2, C. (Institute, City, State, Country). Personal communication, 2012.
6. Author 1, A.B.; Author 2, C.D.; Author 3, E.F. Title of Presentation. In Title of the Collected Work (if available), Proceedings of the Name of the Conference, Location of Conference, Country, Date of Conference; Editor 1, Editor 2, Eds. (if available); Publisher: City, Country, Year (if available); Abstract Number (optional), Pagination (optional).
7. Author 1, A.B. Title of Thesis. Level of Thesis, Degree-Granting University, Location of University, Date of Completion.
8. Title of Site. Available online: URL (accessed on Day Month Year).
