# Supplementary figures and images for: An Ontological Framework to Facilitate Early Detection of ‘Radicalization’ (OFEDR)—A Three World Perspective
Source: J Imaging. 2021 Mar 22;7(3):60. doi: 10.3390/jimaging7030060 (PMC8321290; doi:10.3390/jimaging7030060)

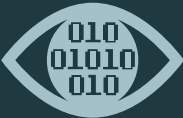

# Journal of *Imaging*

Supplement: Supplementary file 1 [file jimaging-07-00060-s001.zip › jimaging-1113344 final TBS/jimaging-1113344 final/Definitions/jimaging-logo-eps-converted-to.pdf]

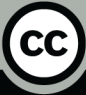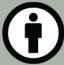

BY

Supplement: Supplementary file 1 [file jimaging-07-00060-s001.zip › jimaging-1113344 final TBS/jimaging-1113344 final/Definitions/logo-ccby-eps-converted-to.pdf]

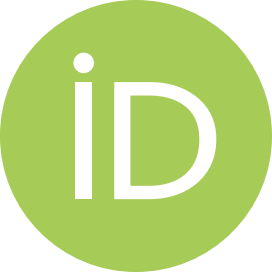

Supplement: Supplementary file 1 [file jimaging-07-00060-s001.zip › jimaging-1113344 final TBS/jimaging-1113344 final/Definitions/logo-orcid-eps-converted-to.pdf]

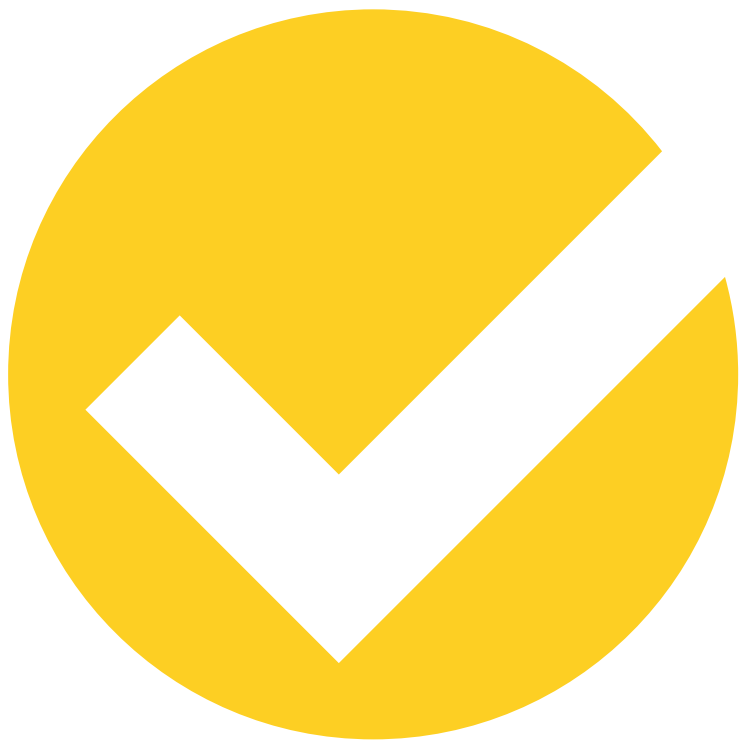

check for  
updates

Supplement: Supplementary file 1 [file jimaging-07-00060-s001.zip › jimaging-1113344 final TBS/jimaging-1113344 final/Definitions/logo-updates.pdf]

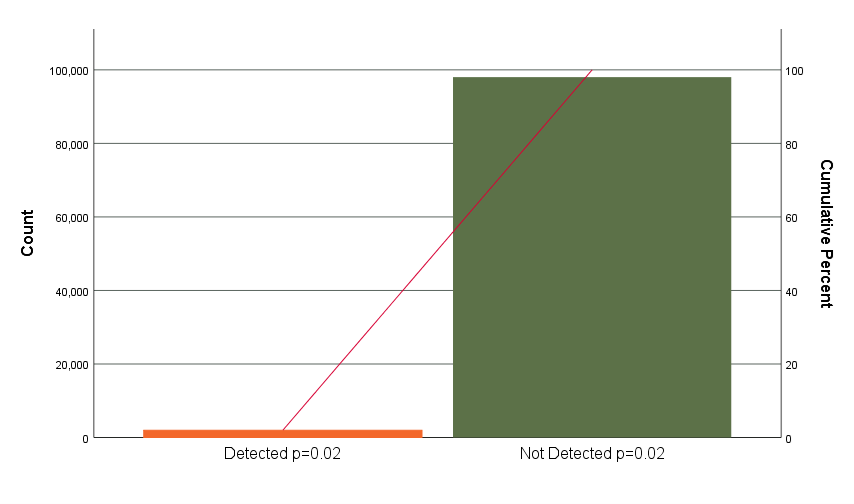

Supplement: Supplementary file 1 [file jimaging-07-00060-s001.zip › jimaging-1113344 final TBS/jimaging-1113344 final/Fig14a_new.png]

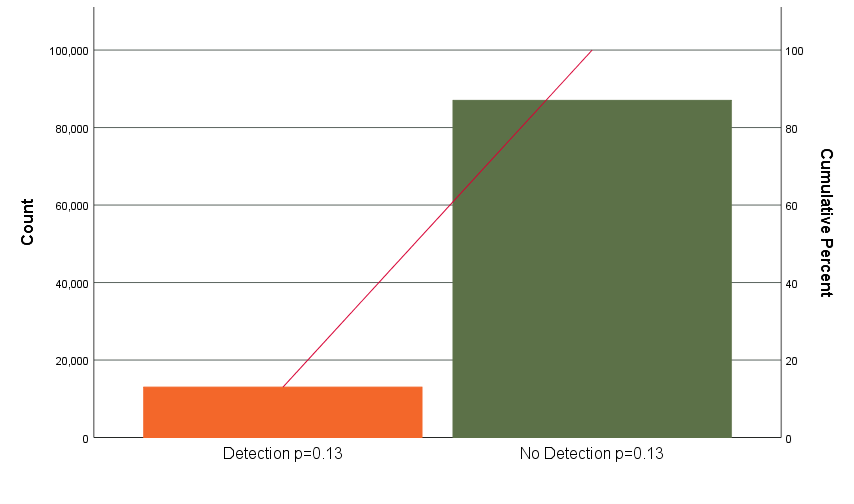

Supplement: Supplementary file 1 [file jimaging-07-00060-s001.zip › jimaging-1113344 final TBS/jimaging-1113344 final/Fig14b_new.png]

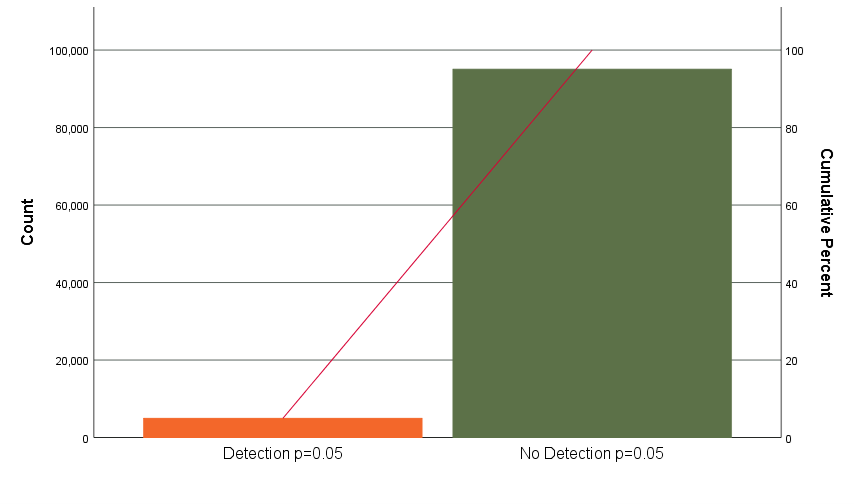

Supplement: Supplementary file 1 [file jimaging-07-00060-s001.zip › jimaging-1113344 final TBS/jimaging-1113344 final/Fig14c_new.png]

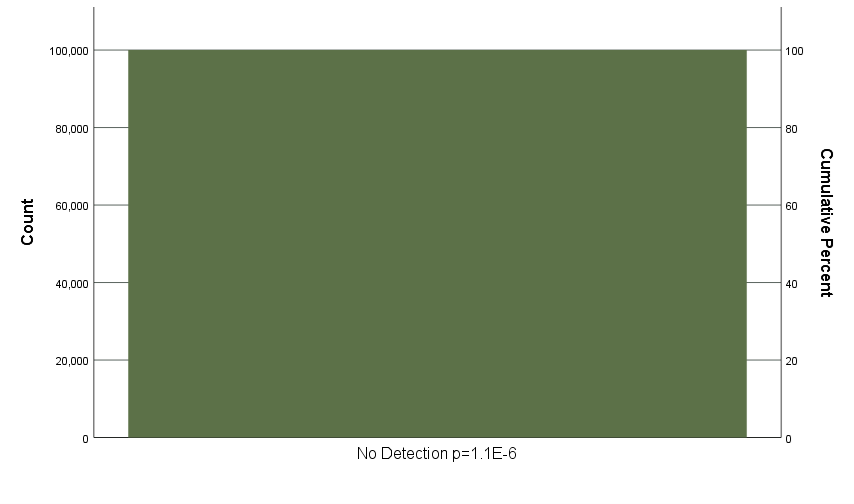

Supplement: Supplementary file 1 [file jimaging-07-00060-s001.zip › jimaging-1113344 final TBS/jimaging-1113344 final/Fig14d_new.png]

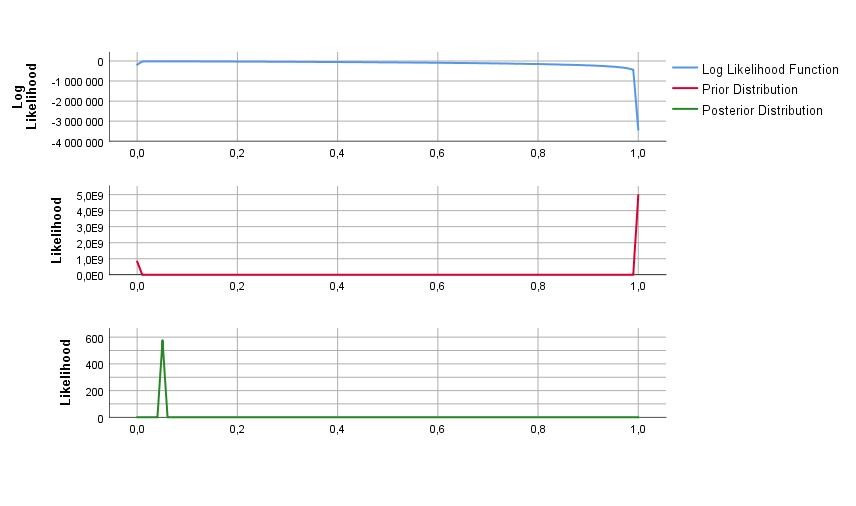

Supplement: Supplementary file 1 [file jimaging-07-00060-s001.zip › jimaging-1113344 final TBS/jimaging-1113344 final/Fig14New.jpg]

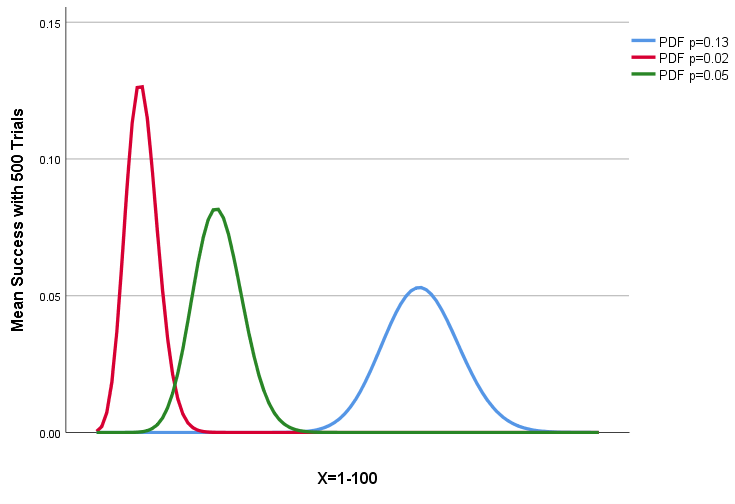

Supplement: Supplementary file 1 [file jimaging-07-00060-s001.zip › jimaging-1113344 final TBS/jimaging-1113344 final/Fig15a_new.png]

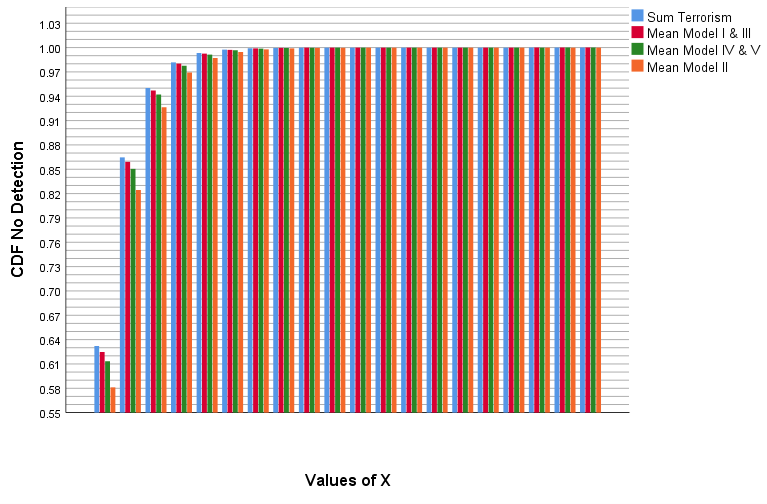

Supplement: Supplementary file 1 [file jimaging-07-00060-s001.zip › jimaging-1113344 final TBS/jimaging-1113344 final/Fig15b_new.png]

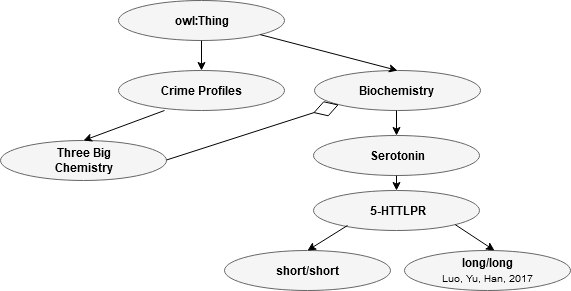

Supplement: Supplementary file 1 [file jimaging-07-00060-s001.zip › jimaging-1113344 final TBS/jimaging-1113344 final/FigurBiochemistry_UsecaseInteraction.png]

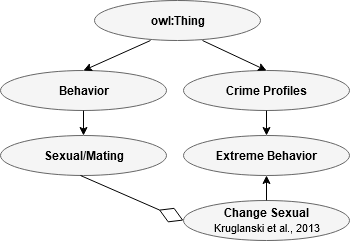

Supplement: Supplementary file 1 [file jimaging-07-00060-s001.zip › jimaging-1113344 final TBS/jimaging-1113344 final/Figure_ChangeSexualRelationshipCrimeProfiles_NEW.png]

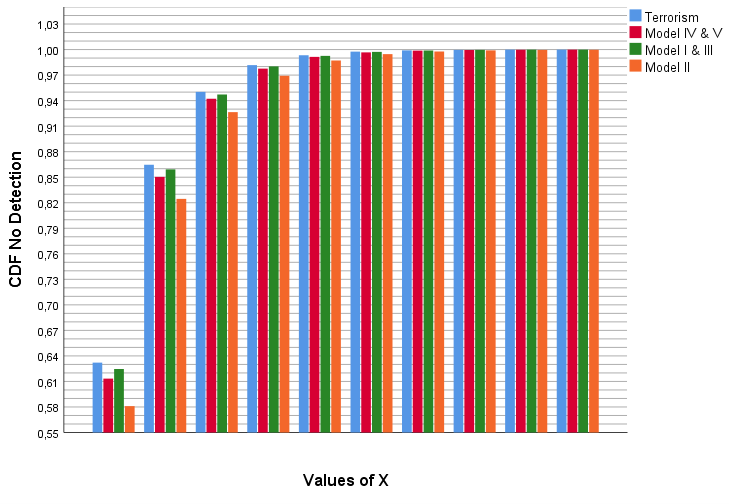

Supplement: Supplementary file 1 [file jimaging-07-00060-s001.zip › jimaging-1113344 final TBS/jimaging-1113344 final/Figure14bHvit.png]

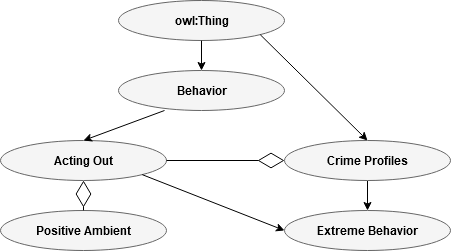

Supplement: Supplementary file 1 [file jimaging-07-00060-s001.zip › jimaging-1113344 final TBS/jimaging-1113344 final/FigureActingOutRelationshipToCrimeProfilesAndAmbient.png]

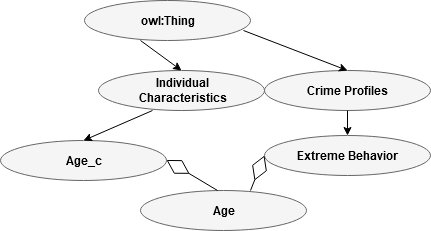

Supplement: Supplementary file 1 [file jimaging-07-00060-s001.zip › jimaging-1113344 final TBS/jimaging-1113344 final/FigureAge_New.png]

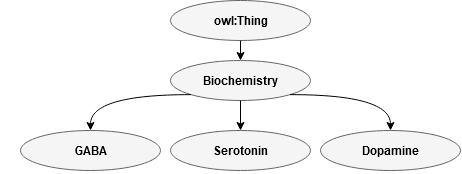

Supplement: Supplementary file 1 [file jimaging-07-00060-s001.zip › jimaging-1113344 final TBS/jimaging-1113344 final/FigureBiochemistry_New.png]

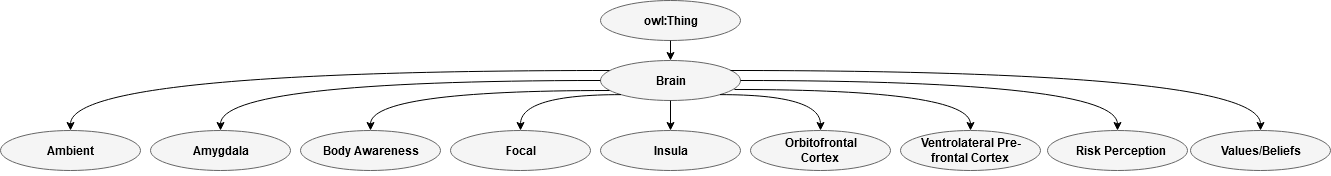

Supplement: Supplementary file 1 [file jimaging-07-00060-s001.zip › jimaging-1113344 final TBS/jimaging-1113344 final/FigureBrainProcessing_New.png]

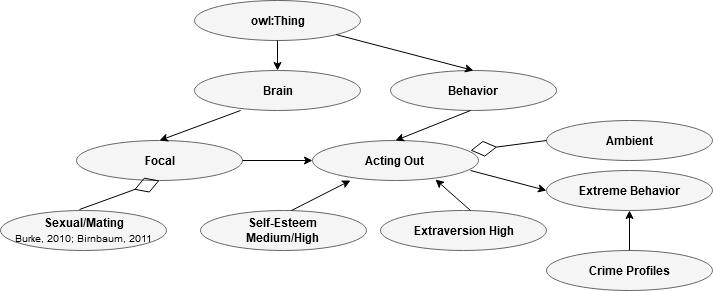

Supplement: Supplementary file 1 [file jimaging-07-00060-s001.zip › jimaging-1113344 final TBS/jimaging-1113344 final/FigureFocalUsercase_New.png]

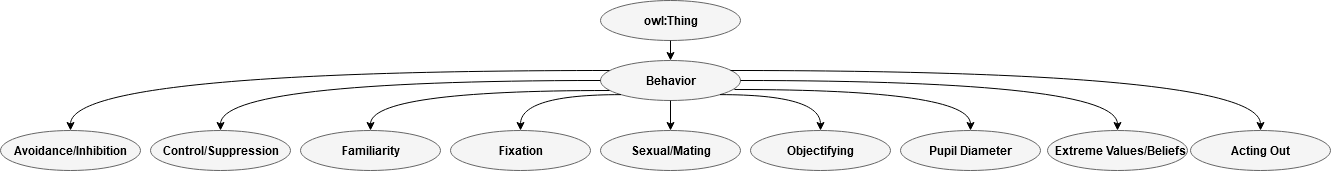

Supplement: Supplementary file 1 [file jimaging-07-00060-s001.zip › jimaging-1113344 final TBS/jimaging-1113344 final/FigureNewBehavior.png]

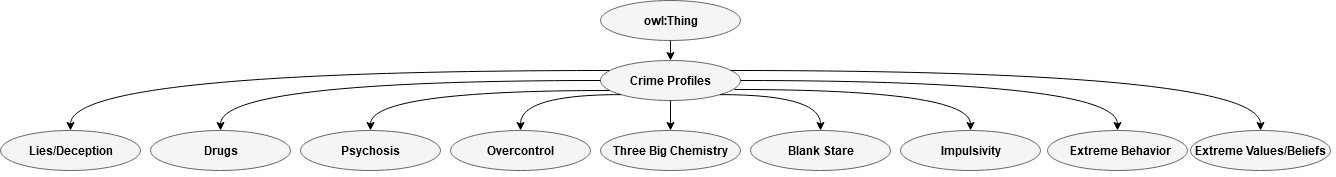

Supplement: Supplementary file 1 [file jimaging-07-00060-s001.zip › jimaging-1113344 final TBS/jimaging-1113344 final/FigureNEwCrimeProfiles.png]

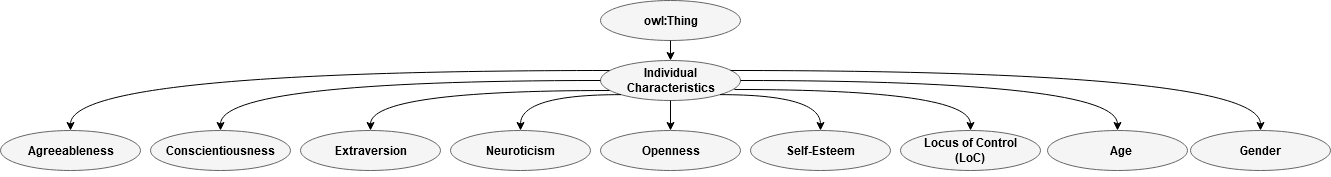

Supplement: Supplementary file 1 [file jimaging-07-00060-s001.zip › jimaging-1113344 final TBS/jimaging-1113344 final/FigurIndividualCharacteristicsNEW.png]

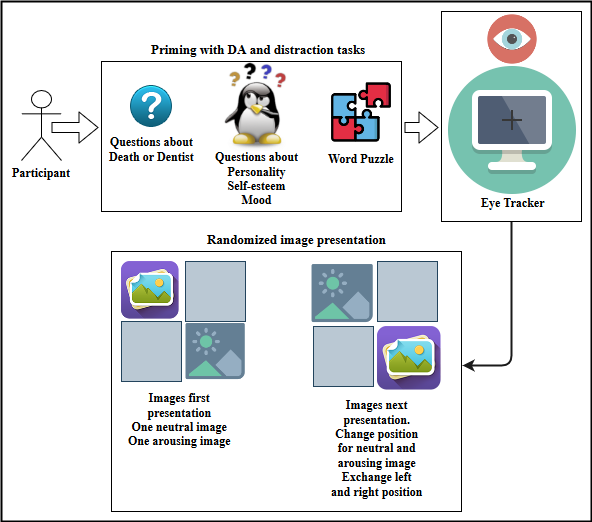

Supplement: Supplementary file 1 [file jimaging-07-00060-s001.zip › jimaging-1113344 final TBS/jimaging-1113344 final/Nedlastet flytdiagram met600dpi.png]

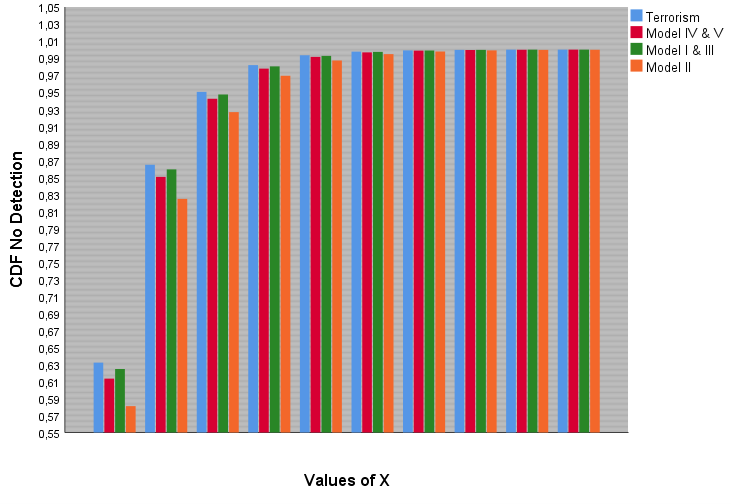

Supplement: Supplementary file 1 [file jimaging-07-00060-s001.zip › jimaging-1113344 final TBS/jimaging-1113344 final/nyFIg15b.png]

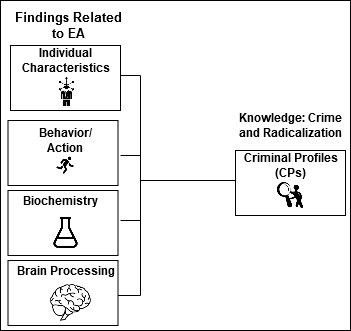

Supplement: Supplementary file 1 [file jimaging-07-00060-s001.zip › jimaging-1113344 final TBS/jimaging-1113344 final/NyFigureOne.png]

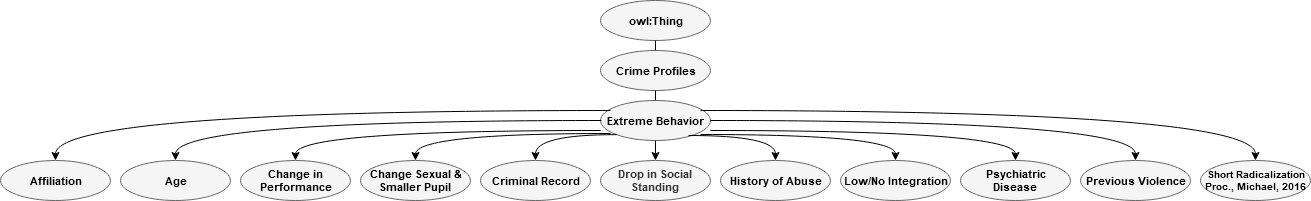

Supplement: Supplementary file 1 [file jimaging-07-00060-s001.zip › jimaging-1113344 final TBS/jimaging-1113344 final/NyFigurExtreme.png]

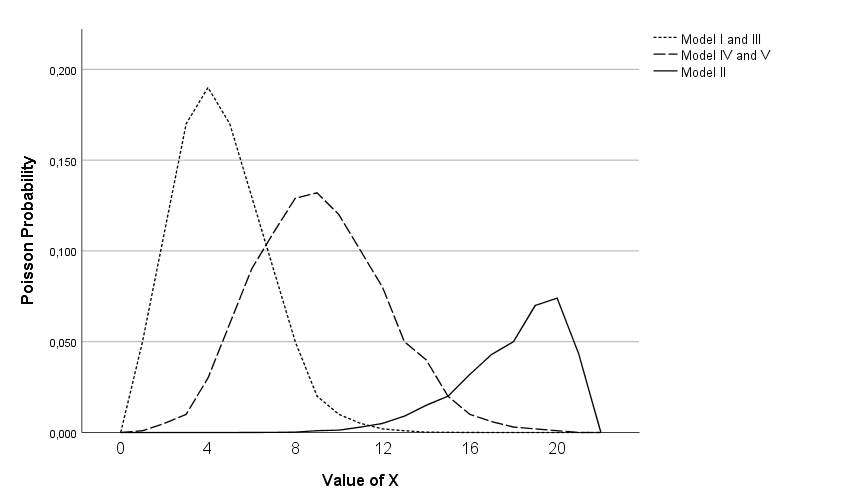

Supplement: Supplementary file 1 [file jimaging-07-00060-s001.zip › jimaging-1113344 final TBS/jimaging-1113344 final/PoissonExModel.png]

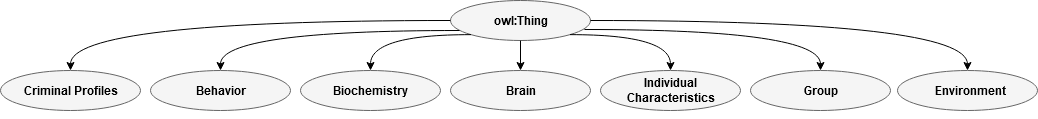

Supplement: Supplementary file 1 [file jimaging-07-00060-s001.zip › jimaging-1113344 final TBS/jimaging-1113344 final/TopHiearchy_newJan21.png]
